# Semantic Content of Visual Consequent Stimuli Modulates Audiovisual Learning in Migraine

**DOI:** 10.1002/brb3.71697

**Published:** 2026-08-20

**Authors:** Noémi Harcsa‐Pintér, Kálmán Tót, Adél Papp, Gabriella Eördegh, Gábor Braunitzer, Balázs Bodosi, Anett Csáti, János Tajti, Attila Nagy

**Affiliations:** ^1^ Department of Physiology, Albert Szent‐Györgyi Medical School University of Szeged Szeged Hungary; ^2^ Department of Theoretical Health Sciences and Health Management, Faculty of Health Sciences and Social Studies University of Szeged Szeged Hungary; ^3^ Sztárai Institute University of Tokaj Sárospatak Hungary; ^4^ Department of Neurology University of Szeged Szeged Hungary

**Keywords:** associative equivalence learning, cognitive functions, human, migraine

## Abstract

**Introduction:**

Acquired equivalence learning relies on associating different antecedent stimuli with shared outcomes and is mediated by the basal ganglia and hippocampus. Audiovisual associative learning benefits from multisensory integration, which may be disrupted in migraine. Previous studies indicated altered performance in such learning paradigms. This study investigated whether the semantic content of consequent visual stimuli influence audiovisual associative equivalence learning in adult migraine patients.

**Methods:**

30 adult migraine patients completed two audiovisual learning tests: the SoundFace test (cartoon faces as visual consequents) and the SoundPolygon test (simple geometric shapes). Both used the same four auditory antecedents. Participants completed acquisition with feedback, retrieval of learned associations, and generalization to novel stimulus combinations. Performance was assessed by the number of trials required to learn, error ratios during each phase, and average response times.

**Results:**

Patients performed significantly better in the SoundFace test than in the SoundPolygon test. They required fewer acquisition trials, committed fewer errors across all phases, and showed shorter reaction times. No significant differences were observed between migraine patients and controls in the SoundPolygon test.

**Conclusion:**

Simpler visual consequents elicited reduced audiovisual learning performance in patients, consistent with a previous control study. However, no impaired learning performance was found relative to controls when reduced visual consequents were applied. Migraine patients outperformed controls in the SoundFace test previously. These findings suggest that while reduced semantic content impairs audiovisual learning, migraine patients show preserved performance compared to controls. The results may reflect compensatory mechanisms preserving cognitive function in migraine.

## Graphical abstract

Adult migraine patients performed better in audiovisual acquired equivalence learning when faces with higher semantic content, rather than simple geometric shapes, served as visual outcomes. However, their performance with geometric shapes did not differ significantly from that of controls. These findings suggest that semantic content facilitates audiovisual associative learning, while cognitive performance remains preserved in migraine.

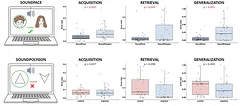

## 1 Introduction

A paradigm to assess acquired equivalence learning is the Rutgers Acquired Equivalence Test (RAET) (Myers et al. 2003), which uses cartoon faces and colored fish as visual stimuli. In this paradigm, participants first learn associations through feedback‐based trial‐and‐error learning, and are then tested on their ability to retrieve and generalize these associations to novel combinations without further feedback (de Araujo Sanchez and Zeithamova 2023; Hall 1996; Meeter et al. 2009).

The underlying neural structures of acquired equivalence learning are well documented: the basal ganglia play a key role in the acquisition phase, while the hippocampus is critical for retrieval and generalization (the two parts of the test phase) (Meeter et al. 2009; Shohamy and Wagner 2008). The distinct involvement of these brain regions in different phases of the task has been demonstrated both in patient studies and in neuroimaging research, supporting the interpretation of acquired equivalence as a form of integrative encoding (de Araujo Sanchez and Zeithamova 2023; Moustafa et al. 2010; Myers et al. 2003; Shohamy and Wagner 2008). Altered sensory processing and cognitive functions are frequently reported during the interictal periods of migraine, affecting both unimodal and multimodal perception (Braganza et al. 2022; Braunitzer et al. 2010; Braunitzer et al. 2012; Braunitzer et al. 2011; Camarda et al. 2007; de Tommaso et al. 2014; Giricz et al. 2021; Huang et al. 2017; Lee et al. 2017; McKendrick and Sampson 2009; Shepherd et al. 2012; Vuralli et al. 2018; Öze et al. 2017). Previous research by our group has demonstrated that adult migraine patients show impaired performance in both phases of the unimodal, visual RAET (Giricz et al. 2021; Öze et al. 2017).

The neural structures involved in acquired equivalence learning—the striatum and the hippocampi—contribute to multisensory processing (Bates and Wolbers 2014; Nagy et al. 2006; Nagy et al. 2005), and both have been implicated in the altered sensory integration observed in migraine (Ashina et al. 2021; Chen et al. 2022; Filippi and Messina 2019; Liu et al. 2018; Liu et al. 2017; Maleki et al. 2011). There is an overlap in the involvement of hippocampal and basal ganglia circuits in both migraine pathophysiology (Chong et al. 2017; Liu et al. 2018; Maleki et al. 2013) and acquired equivalence learning, including multisensory processes. Multisensory integration involves the convergence and joint processing of inputs from different sensory modalities, and has been shown to enhance learning and memory under certain conditions (Eördegh, Tót, Kiss, et al. 2022; Heikkilä et al. 2015; Xie et al. 2017).

Based on the original RAET paradigm, we previously developed a multisensory adaptation called the SoundFace test (Eördegh et al. 2019), in which auditory antecedents are paired with visual consequents. Performance was not significantly different in the visual RAET compared to the audiovisual SoundFace test among healthy adults (Eördegh et al. 2019). In contrast, in a healthy pediatric population, we found that audiovisual pair learning was more efficient than unimodal visual learning, supporting the facilitating role of multisensory integration (Eördegh, Tót, Kiss, et al. 2022). However, this benefit was not as clearly observed in children with migraine, indicating a possible disruption of audiovisual learning mechanisms in this population (Braunitzer et al. 2023). Furthermore, our latest finding showed that adult migraine patients performed significantly better in the acquisition phase of the SoundFace test compared to healthy adults, suggesting a possible, hitherto unknown compensatory mechanism in the cognitive processes of migraine patients (Tót et al. 2024).

Semantic content and stimulus complexity (e.g., number of features) are known to influence learning and memory processes (Arslan et al. 2020; Ashby et al. 1998; Ashby and Maddox 2005; Zettersten and Lupyan 2020). To investigate this effect, we developed the SoundPolygon test by reducing the semantic content of the visual consequent stimuli (Tót et al. 2022). We used the same auditory antecedent stimuli as in the SoundFace test, but instead of cartoon faces, we applied simple geometric shapes as consequents.

Previous findings using this audiovisual acquired equivalence paradigm suggest that reducing the semantic content of the visual stimuli worsens acquisition, retrieval, and generalization performance in healthy adults compared to the SoundFace test, which applies visual stimuli with higher semantic content (Tót et al. 2022).

The primary aim of this study was to compare audiovisual acquired equivalence learning within a migraine patient group using the SoundFace and SoundPolygon tests that differ only in the semantic content of the visual consequent stimuli. To facilitate the interpretation of the results, we additionally compared learning performance in the SoundPolygon test between adult migraine patients and matched healthy controls.

## 2 Methods

### 2.1 Participants

Participants were recruited on a voluntary basis, patients from the Department of Neurology, Albert Szent‐Györgyi Health Center, University of Szeged, Hungary. All participants provided written informed consent. The inclusion criterion for patients was a diagnosis of migraine without aura, established by neurologists according to the ICHD‐3 criteria. Exclusion criteria for both the patient group and the control group included any history of neurological, psychiatric, ophthalmologic, or otologic disorders. Participants reporting memory impairment, learning difficulties, or any conditions potentially affecting cognitive performance were also excluded from the study. All testing sessions for patients were conducted during the interictal period, ensuring that at least three days had passed since the participants’ last migraine attack.

Migraine participants reported a mean attack frequency of 3.1 attacks per month, an estimated lifetime total of approximately 400–500 migraine attacks, and a mean migraine history of 12.4 years (149.2 months), based on participants’ self‐reported symptom onset.

Patients receiving preventive (interval) migraine therapy or medications known to affect central nervous system (e.g., antidepressants or topiramate) were excluded from the study. The use of acute migraine medication (e.g., triptans) during attacks was permitted.

Initially, 43 patients were recruited, of whom 30 met the inclusion criteria, completed the full study protocol, and were included in the final analysis. We matched 30 healthy controls to the patient group based on age and level of education.

The study protocol was conducted in accordance with the Declaration of Helsinki and received approval from the Regional Research Ethics Committee for Medical Research at the University of Szeged, Hungary (Approval number: 27/2020‐SZTE, NNGYK/31295‐6/2024).

### 2.2 Tests and Procedures

The tests were administered individually on a personal computer equipped with a CRT monitor (refresh rate: 60 Hz). Participants were seated at a comfortable distance from the screen and used a joystick with left and right buttons to register their responses. All sessions took place in a quiet room to minimize distractions and facilitate focused participation. To reduce carry‐over effects, tests were presented in a counterbalanced order.

The SoundFace and SoundPolygon tests, modified audiovisual versions of the original Rutgers Acquired Equivalence Test (RAET) by Myers and colleagues (Myers et al. 2003), were employed (Eördegh et al. 2019; Tót et al. 2022). In these audiovisual adaptations, participants learned to associate visual stimuli (cartoon faces in SoundFace and geometric polygons in SoundPolygon) with the same four everyday sounds (e.g., vehicle noise, guitar strum, human voice, or cat's meow). During each trial, participants heard one of the four sounds and had to choose between two visual stimuli, identifying the face or polygon corresponding to the presented sound.

Both tests consisted of two main phases: acquisition phase and test phase.

During acquisition, participants learned association pairs through immediate feedback. In each trial, a single antecedent (one of the four sounds mentioned above) was played, while two consequent options (cartoon faces in the SoundFace test and two‐dimensional blank geometric shapes in the SoundPolygon test) were shown on the left and right sides of the screen. Participants selected the corresponding consequent by pressing either the left or right button on the joystick.

**FIGURE 1:**
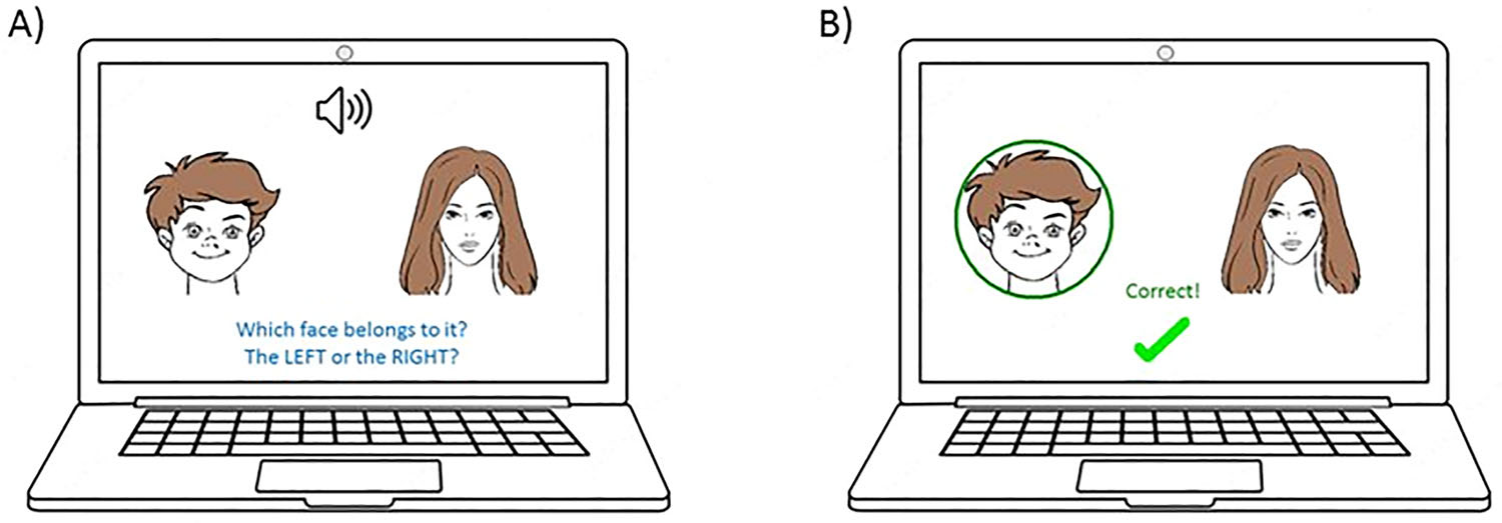
(A) Example of a trial in the acquisition phase of the SoundFace test. Participants hear an antecedent sound (vehicle noise, guitar strum, human voice, or cat's meow) and see two visual consequent stimuli (cartoon faces) on the left and right sides of the screen. They must choose which of the two consequents corresponds to the given antecedent by pressing the left or right button on the joystick. (B) Visual feedback (a green checkmark and the word “Correct!”) following a correct response.

**FIGURE 2:**
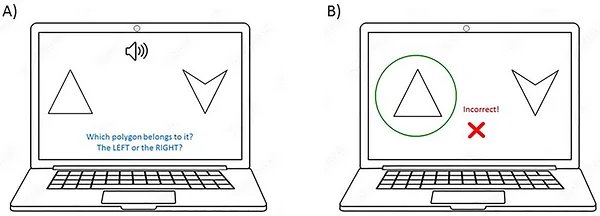
(A) Example of a trial in the acquisition phase of the SoundPolygon test. Participants hear an antecedent sound (vehicle noise, guitar strum, human voice, or cat's meow) and see two visual consequent stimuli (two‐dimensional blank geometric shapes) on the left and right sides of the screen. They must choose which of the two consequents corresponds to the given antecedent by pressing the left or right button on the joystick. (B) Visual feedback (a red X and the word “Incorrect!”) following an incorrect response.

On first exposure to a stimulus pair, the participant's choice was necessarily random. Following their choice, immediate feedback was provided: a green checkmark and the sign “Correct!” for correct responses (Figure 1), or a red “X” and the sign “Incorrect!” for incorrect responses (Figure 2). This feedback allowed participants to learn the correct associations.

Stimulus pairs were introduced gradually, one at a time. After each new pair was introduced, participants were required to achieve a specified number of consecutive correct responses (4, 6, 8, 10, or 12, depending on the sequence) before advancing.

Initially, two association pairs were presented (A1: X1 and B1: Y1). Subsequently, new antecedents were introduced that shared consequents with the initial pairs (A2: X1 and B2: Y1), establishing equivalence (A1 = A2, B1 = B2). In the final stage of acquisition, additional associations (A1: X2 and B1: Y2) were introduced. Of the eight possible association pairs, six were taught to each participant.

**FIGURE 3:**
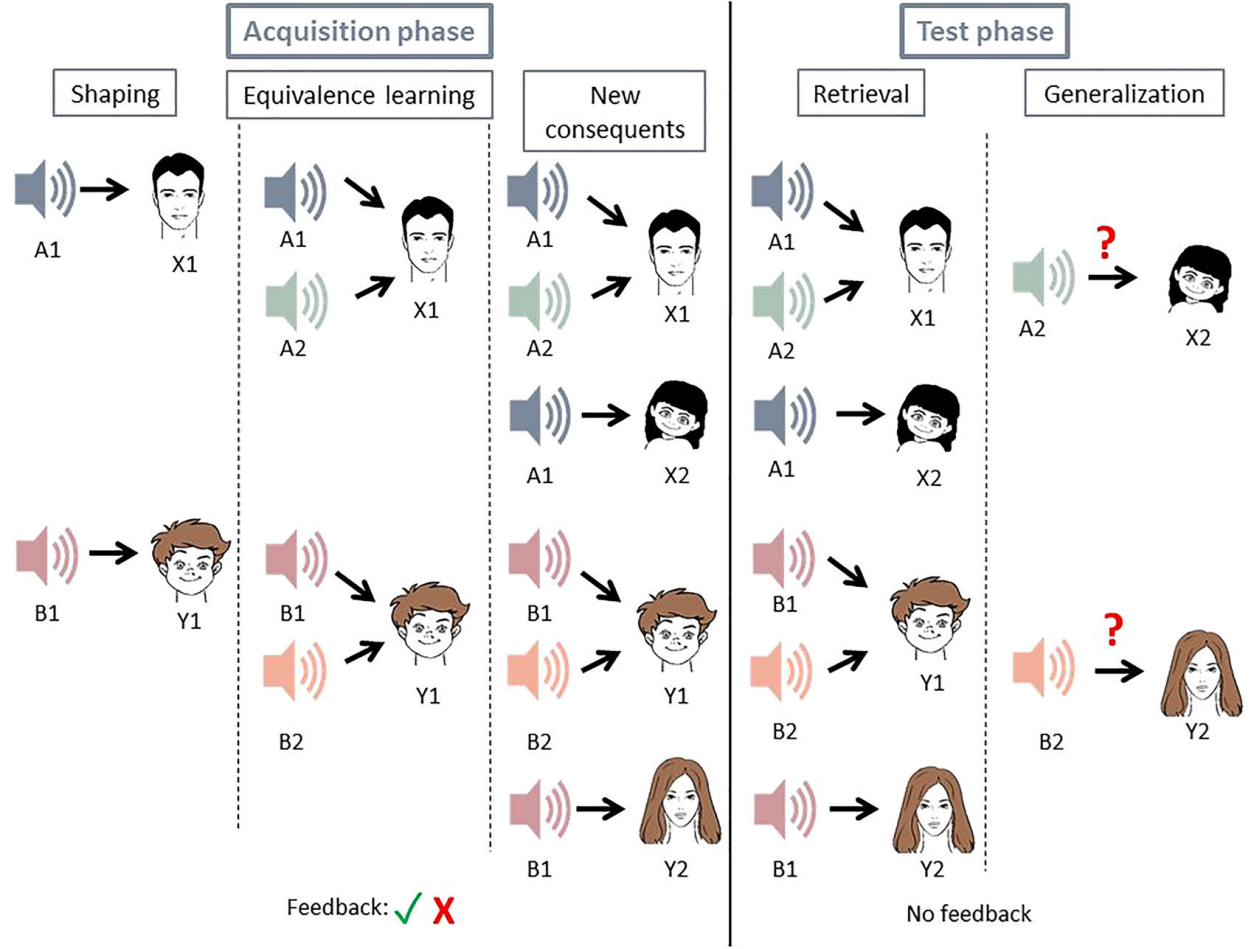
Overview of the structure of the SoundFace test. In the acquisition phase, participants gradually learn which face belongs to the presented sound (shaping) based on feedback. Equivalence is formed between two sound stimuli due to a shared consequence face stimulus. A new consequent face stimulus is also associated to one of the equivalent antecedent sounds. In the test phase, feedback is not provided. Participants have to recall previously learned pairs and apply the equivalence to novel stimulus pairs (generalization).

**FIGURE 4:**
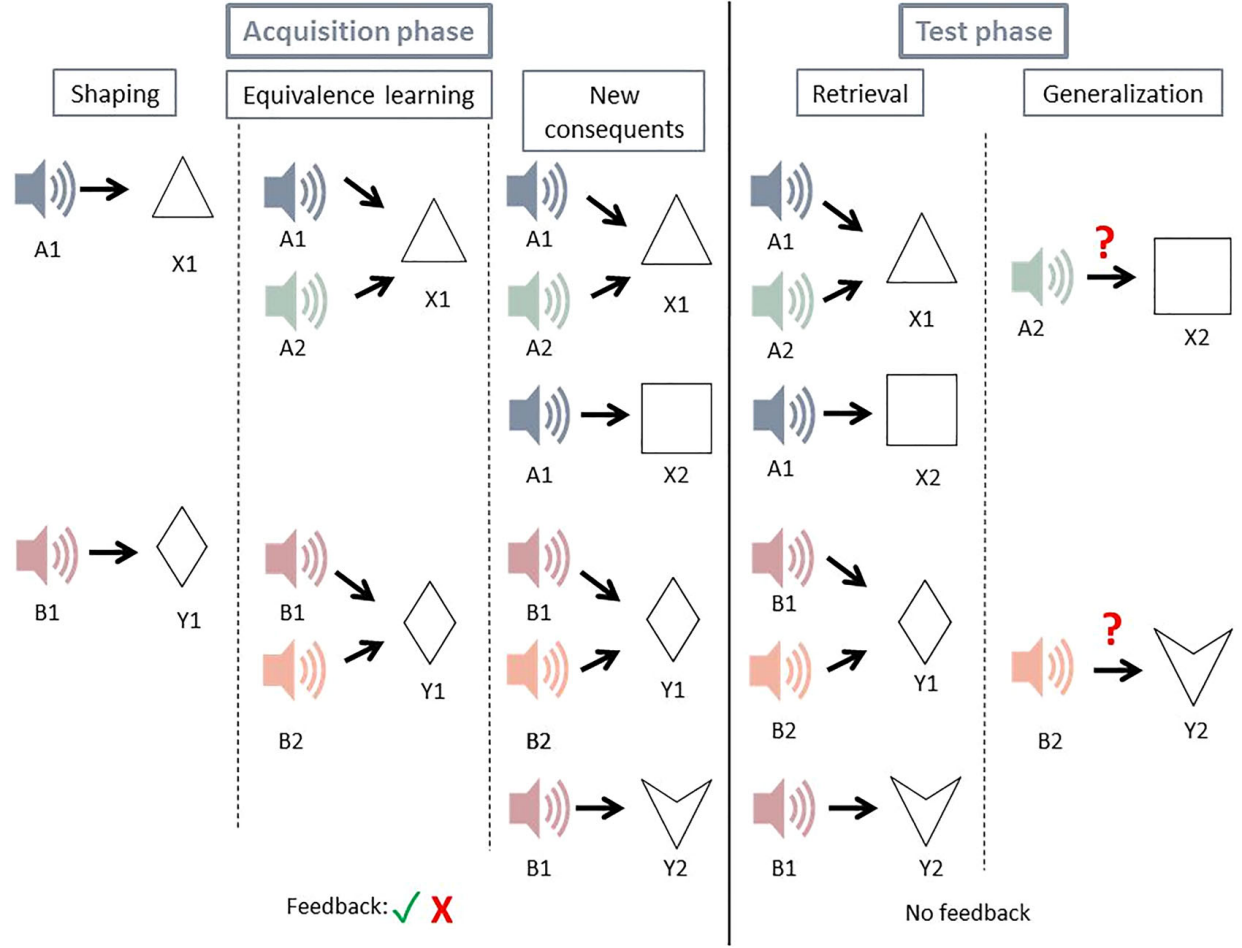
Overview of the structure of the SoundPolygon test. In the acquisition phase, participants gradually learn which polygon belongs to the presented sound (shaping) based on feedback. Equivalence is formed between two sound stimuli due to a shared consequence polygon stimulus. A new consequent polygon stimulus is also associated to one of the equivalent antecedent sounds. In the test phase, feedback is not provided. Participants have to recall previously learned pairs and apply the equivalence to novel stimulus pairs (generalization).

In the subsequent test phase, no feedback was provided. Participants were required to recall the previously learned six associations (retrieval) and apply the acquired equivalence to infer associations for two previously unseen pairs (generalization: A2: X2 and B2: Y2; Figures 3 and 4).

The number of trials in the test phase was fixed, consisting of 36 retrieval trials and 12 generalization trials presented in random order.

### 2.3 Data Analysis

Number of Acquisition Trials (NAT), defined as the total number of trials needed to learn the associations; Acquisition Error Ratio (AER), the proportion of errors during acquisition; Retrieval Error Ratio (RER), the proportion of errors during retrieval; and Generalization Error Ratio (GER), the proportion of errors during generalization were calculated. Reaction times (RTs) were also recorded in milliseconds for each trial and averaged separately for the acquisition, retrieval, and generalization phases.

Statistical analyses were conducted using Statistica 14.0.0.15 (TIBCO Software Inc.) and G*Power (version 3.1.9.7). The Shapiro–Wilk test was applied to assess the normality of distributions. As most data were non‐normally distributed, Wilcoxon matched‐pairs tests were used to compare performances and reaction times between the SoundFace and SoundPolygon tests within the migraine group. Effect sizes were reported using rank‐biserial correlation (*r*), where *r* = *Z*/√*N* (*Z*: result of the statistical test, *N*: valid sample size). For the normally distributed data (acquisition reaction time, ART) paired *t*‐test was used, and effect size was calculated as *dz* = *T*/√*N* (*T*: result of the statistical test, *N*: valid sample size). Mann–Whitney *U* test was used to compare performances of the patient group and the healthy control group. Effect sizes were calculated the same way as in the within subject analyses.

An a priori sample size estimation was performed before data collection using G*Power 3.1.9.7. Assuming *α* = 0.05, statistical power (1 − *β*) = 0.80, and an expected effect size of *dz* = 0.5, the required sample size was estimated to be 28 participants for the within‐group comparisons (Wilcoxon matched‐pairs test) and 53 participants per group for the between‐group comparisons (Mann–Whitney *U* test).

## 3 Results

Of the 43 migraine patients recruited, 13 did not meet the inclusion criteria; therefore, we analyzed and present the results of the remaining 30 participants.

### 3.1 Comparison of Performance of Migraine Patients in the Acquisition Phase of the Two Audiovisual Tests

**FIGURE 5:**
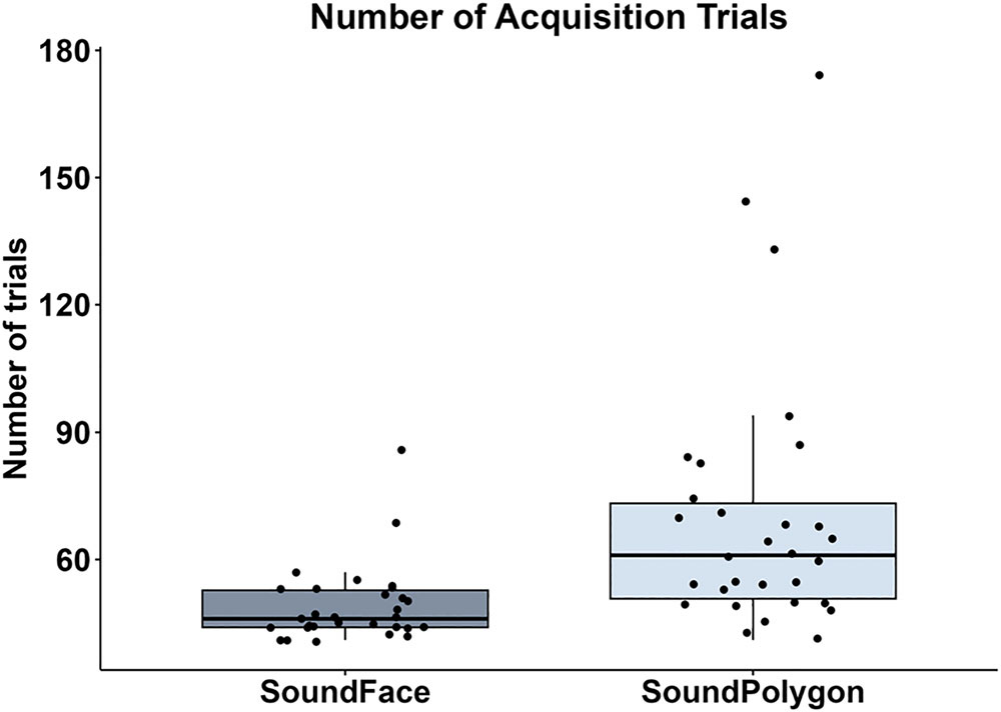
Boxplot comparison of the number of acquisition trials (NAT) required by migraine patients to complete the acquisition phase in the SoundFace and SoundPolygon tests. The upper and lower edges of each box represent the third and first quartiles, respectively. The line within each box indicates the median. Whiskers extend to the largest and smallest data points within 1.5 times the interquartile range (IQR) from the quartiles. Each dot represents an individual data point; those beyond the whiskers are considered outliers. Significantly less trials were required in the SoundFace test (Wilcoxon matched‐pairs test: *n* = 30; *p* < 0.001; *r* = 0.683).

The patients required significantly more trials to learn the associations in the SoundPolygon test than in the SoundFace test (*Z* = 3.676, *p* < 0.001, *r* = 0.683). The median number of acquisition trials (NAT) in the SoundFace test was 46 (Q1: 44–Q3: 53), while in the SoundPolygon test it was 61 (Q1: 50–Q3: 74) (Figure 5).

**FIGURE 6:**
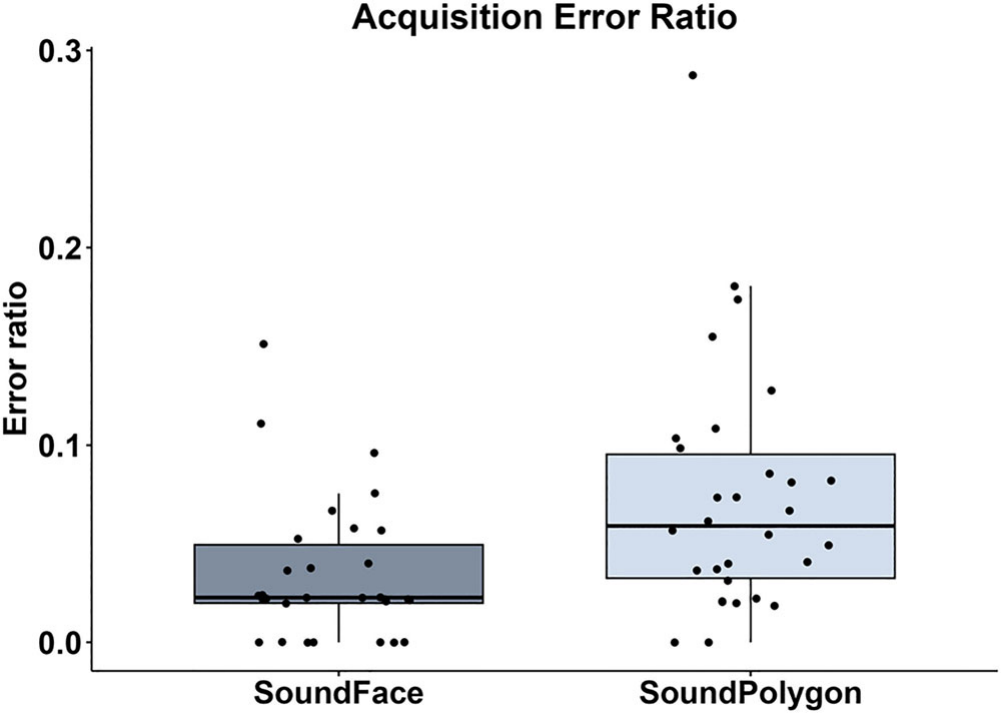
Boxplot comparison of the acquisition error ratios (AER) between the SoundFace and SoundPolygon tests within the migraine patient group. Significantly lower error ratios were found in the SoundFace test (Wilcoxon matched‐pairs test: *n* = 30; *p* < 0.001; *r* = 0.624). Plotting conventions are the same as described in Figure 5.

The acquisition error ratios (AER), calculated by dividing the number of incorrect responses by the total number of acquisition trials, were also significantly lower in the SoundFace test than in the SoundPolygon test (*Z* = 3.362, *p* < 0.001, *r* = 0.624). The median AER was 0.023 in the SoundFace test (Q1: 0.020–Q3: 0.053) and 0.059 in the SoundPolygon test (Q1: 0.031–Q3: 0.099) (Figure 6).

**FIGURE 7:**
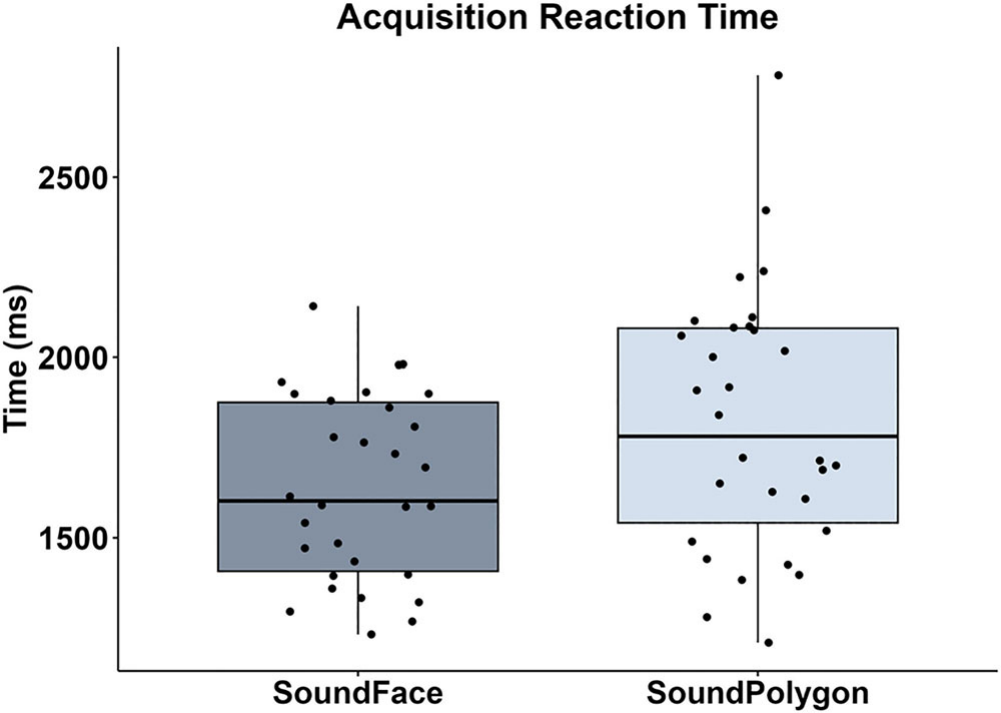
Boxplot comparison of the reaction times between the SoundFace and SoundPolygon tests in the acquisition phase within the migraine patient group. Significantly shorter reaction times were found in the SoundFace test (paired *t*‐test: *n* = 30; *p* = 0.001; *dz* = 0.664). Plotting conventions are the same as described in Figure 5.

The reaction times for the acquisition trials (ART)—defined as the time between the appearance of the auditory and visual stimuli and the participant's response—were significantly shorter in the SoundFace test (*T* = –3.634, *p* = 0.001, *dz* = 0.664). The mean ART in the SoundFace test was 1639 ms (SD: 255 ms), while in the SoundPolygon test it was 1824 ms (SD: 363 ms) (Figure 7).

### 3.2 Comparison of Performance of Migraine Patients in the Test Phase of the Two Audiovisual Tests

**FIGURE 8:**
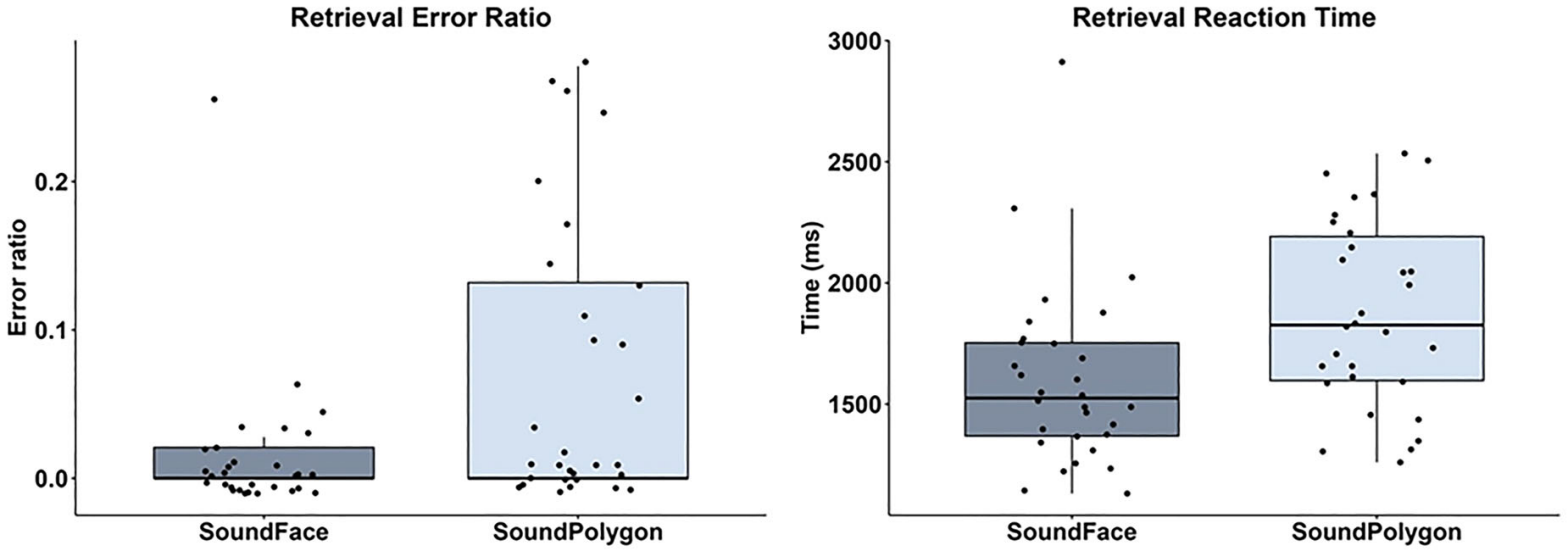
Boxplot comparisons of retrieval performance in the test phase of the SoundFace and SoundPolygon tests within the migraine patient group. Significantly lower error ratios (Wilcoxon matched‐pairs test: *n* = 30; *p* = 0.003; *r* = 0.743 (left)) and significantly shorter reaction times (Wilcoxon matched‐pairs test: *n* = 30; *p* = 0.001; *r* = 0.584 (right)) were found in the SoundFace test. Plotting conventions are the same as described in Figure 5.

The retrieval error ratios (RER), calculated by dividing the number of incorrect responses by the total number of retrieval trials, were significantly lower in the SoundFace test than in the SoundPolygon test (*Z* = 2.973, *p* = 0.003, *r* = 0.743). The median RER in the SoundFace test was 0.000 (Q1: 0.000–Q3: 0.028), while in the SoundPolygon test it was 0.000 (Q1: 0.000–Q3: 0.139). Reaction times during retrieval were also significantly shorter in the SoundFace test (*Z* = 3.198, *p* = 0.001, *r* = 0.584). The median retrieval reaction time (RRT) was 1525 ms in the SoundFace test (Q1: 1366 ms–Q3: 1754 ms) and 1826 ms in the SoundPolygon test (Q1: 1592 ms–Q3: 2207 ms) (Figure 8).

**FIGURE 9:**
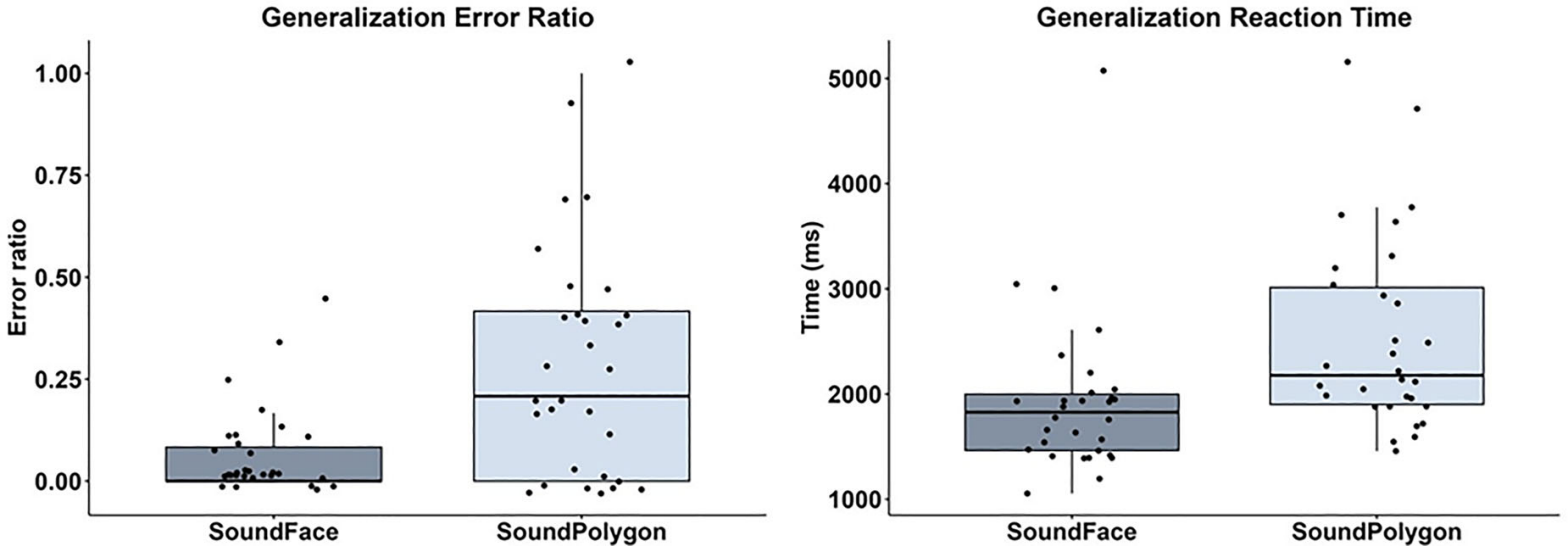
Boxplot comparisons of generalization performance in the test phase of the SoundFace and SoundPolygon tests within the migraine patient group. Significantly lower error ratios (Wilcoxon matched‐pairs test: *n* = 30; *p* < 0.001; *r* = 0.767 (left)) and significantly shorter reaction times (Wilcoxon matched‐pairs test: *n* = 30; *p* = 0.005; *r* = 0.509 (right)) were found in the SoundFace test. Plotting conventions are the same as described in Figure 5.

The generalization error ratios (GER), calculated by dividing the number of incorrect responses by the total number of generalization trials, were also significantly lower in the SoundFace test compared to the SoundPolygon test (*Z* = 3.680, *p* < 0.001, *r* = 0.767). The median GER was 0.000 in the SoundFace test (Q1: 0.000–Q3: 0.083) and 0.208 in the SoundPolygon test (Q1: 0.000–Q3: 0.417). Generalization reaction times (GRT) were also significantly shorter in the SoundFace test than in the SoundPolygon test (*Z* = 2.787, *p* = 0.005, *r* = 0.509). The median GRT in the SoundFace test was 1827 ms (Q1: 1462 ms–Q3: 2013 ms), and in the SoundPolygon test it was 2178 ms (Q1: 1883 ms–Q3: 3039 ms) (Figure 9).

### 3.3 Acquisition Performance of Migraine Patients Compared to That of Healthy Controls in the SoundPolygon Test

**FIGURE 10:**
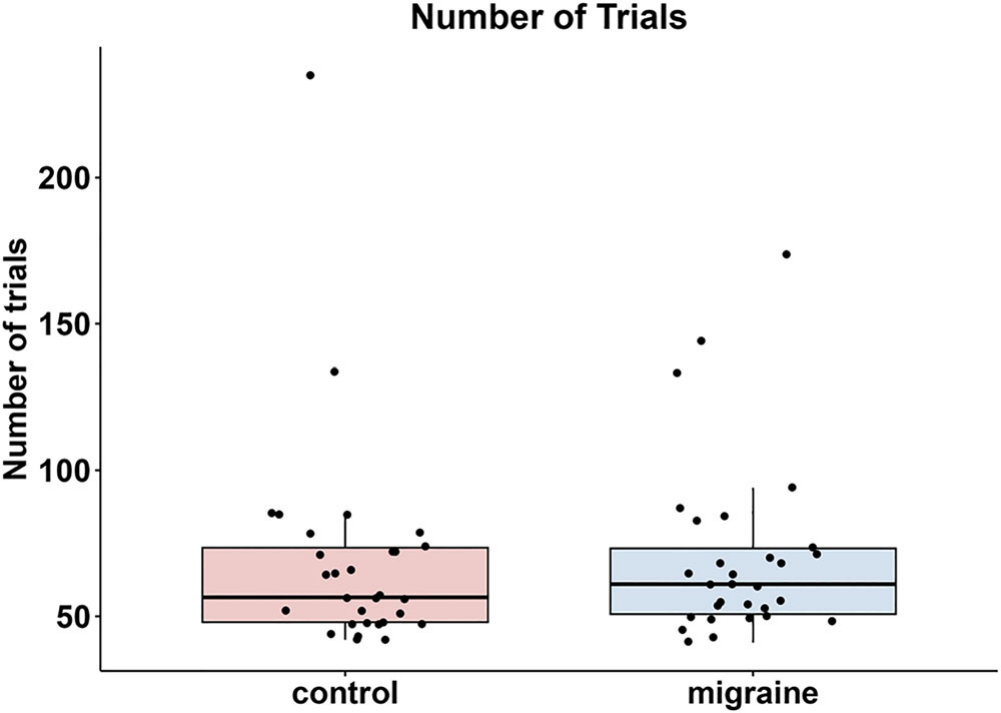
Boxplot comparison of the number of acquisition trials (NAT) required to complete the acquisition phase in the SoundPolygon test in the healthy control group (*n* = 30) and the migraine group (*n* = 30). No significant differences were found between the two groups (Mann–Whitney *U* test: *p* = 0.626; *r* = 0.063). Plotting conventions are the same as described in Figure 5.

There was no significant difference in the number of acquisition trials (NAT) between the patient group and the control group (*U* = 416.50, *p* = 0.626, *r* = 0.063). The median NAT in the patient group was 61 (Q1: 50–Q3: 74), while in the control group, it was 56.5 (Q1: 48–Q3: 74) (Figure 10).

**FIGURE 11:**
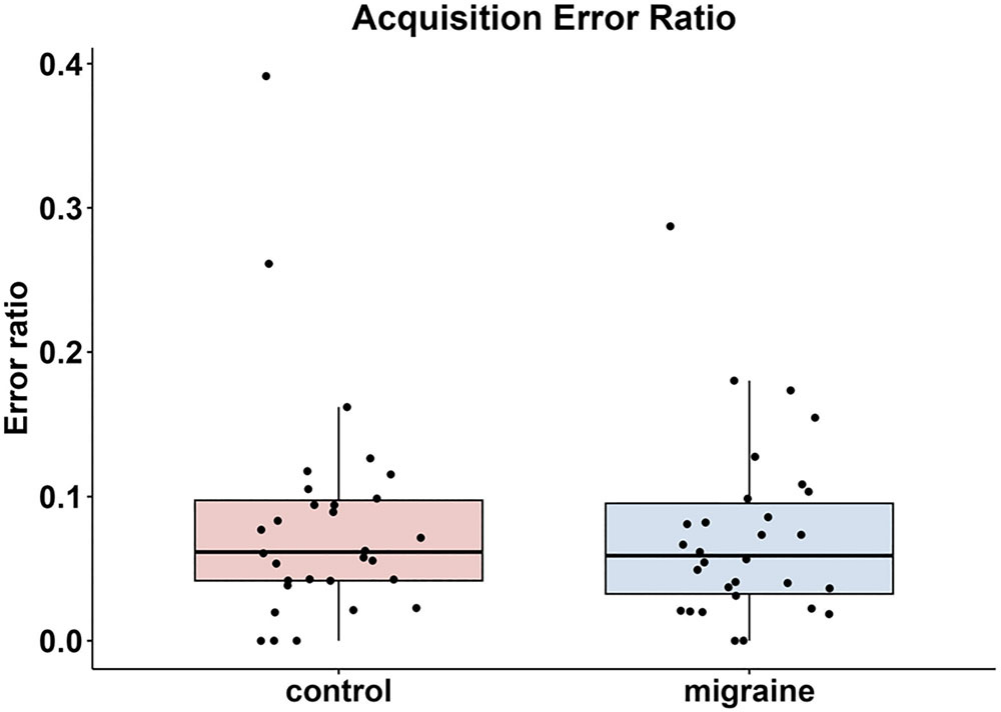
Boxplot comparison of the acquisition error ratios (AER) between the healthy control group (*n* = 30) and the migraine group (*n* = 30) in the SoundPolygon test. No significant differences were found between the two groups (Mann–Whitney *U* test: *p* = 0.657; *r* = 0.057). Plotting conventions are the same as described in Figure 5.

The acquisition error ratios (AER) also did not differ significantly between the two groups (*U* = 419.50, *p* = 0.657, *r* = 0.057). The median AER was 0.059 in the patient group (Q1: 0.031–Q3: 0.099) and 0.062 in the control group (Q1: 0.042–Q3: 0.099) (Figure 11).

**FIGURE 12:**
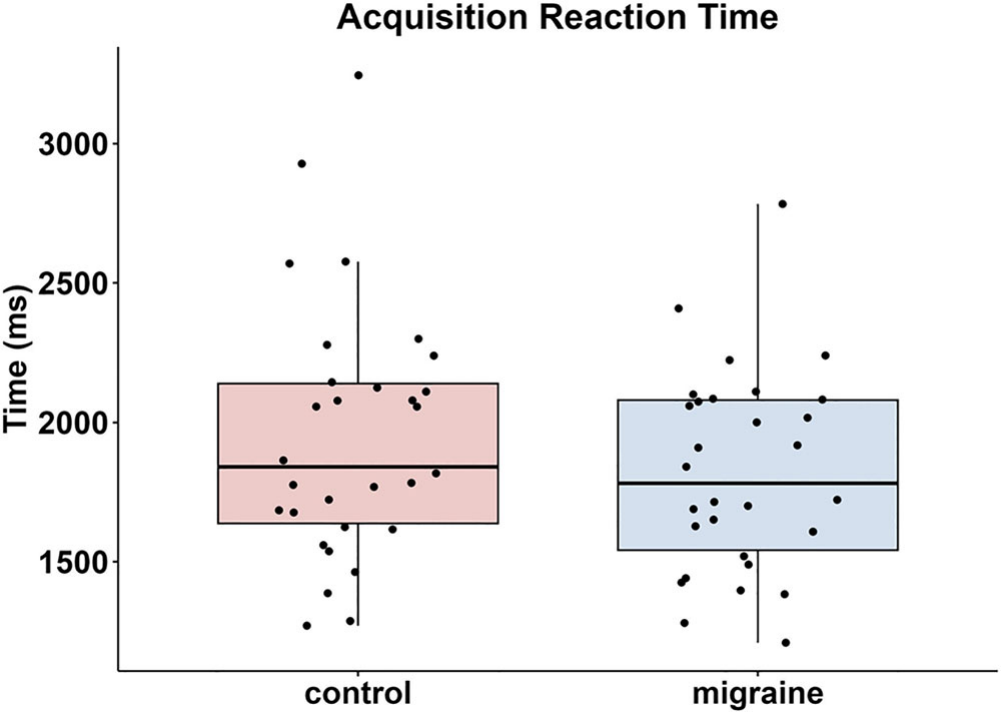
Boxplot comparison of the reaction times between the healthy control group (*n* = 30) and the migraine group (*n* = 30) in the acquisition phase of the SoundPolygon test. No significant differences were found between the two groups (Mann–Whitney *U* test: *p* = 0.290; *r* = 0.136). Plotting conventions are the same as described in Figure 5.

There was no significant difference in the reaction times for the acquisition trials (ART) between the two groups (*U* = 378.00, *p* = 0.290, *r* = 0.136). The median ART in the patient group was 1782 ms (Q1: 1520 ms–Q3: 2083 ms), while in the control group, it was 1841 ms (Q1: 1624 ms–Q3: 2145 ms) (Figure 12).

### 3.4 Retrieval and Generalization Performance of Migraine Patients Compared to Those of Healthy Controls in the SoundPolygon Test

**FIGURE 13:**
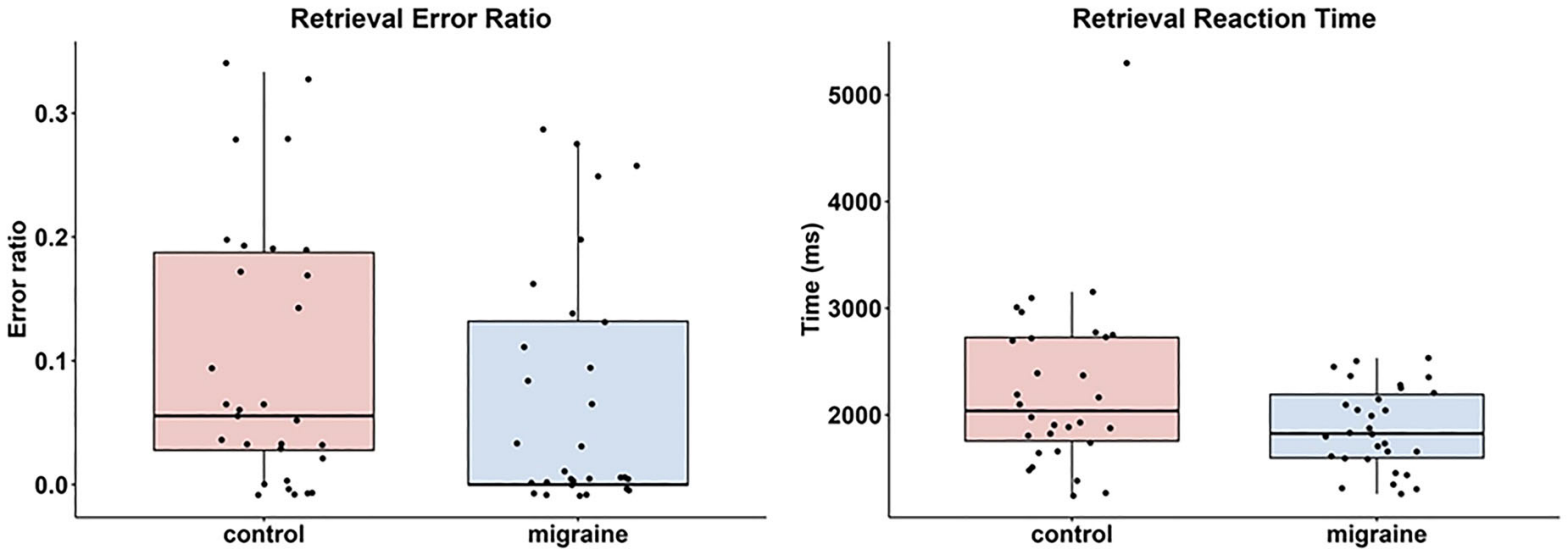
Boxplot comparisons of retrieval performance in the test phase of the SoundPolygon test in the healthy control group (*n* = 30) and the migraine group (*n* = 30). No significant differences were found between the two groups (Mann–Whitney *U* test: *p* = 0.098; *r* = 0.214 for retrieval error ratios (right); and *p* = 0.050; *r* = 0.253 for retrieval reaction times (right)). Plotting conventions are the same as described in Figure 5.

The retrieval error ratios (RER) also did not differ significantly between the patient group and the control group (*U* = 337.50, *p* = 0.098, *r* = 0.214). The median RER in the patient group was 0.000 (Q1: 0.000–Q3: 0.139), while in the control group it was 0.056 (Q1: 0.028–Q3: 0.194). There were no significant differences in reaction times during retrieval (RRT) between the two groups (*U* = 317.00, *p* = 0.050, *r* = 0.253). The median RRT was 1826 ms in the patient group (Q1: 1592 ms–Q3: 2207 ms) and 2039 ms in the control group (Q1: 1739 ms–Q3: 2730 ms) (Figure 13).

**FIGURE 14:**
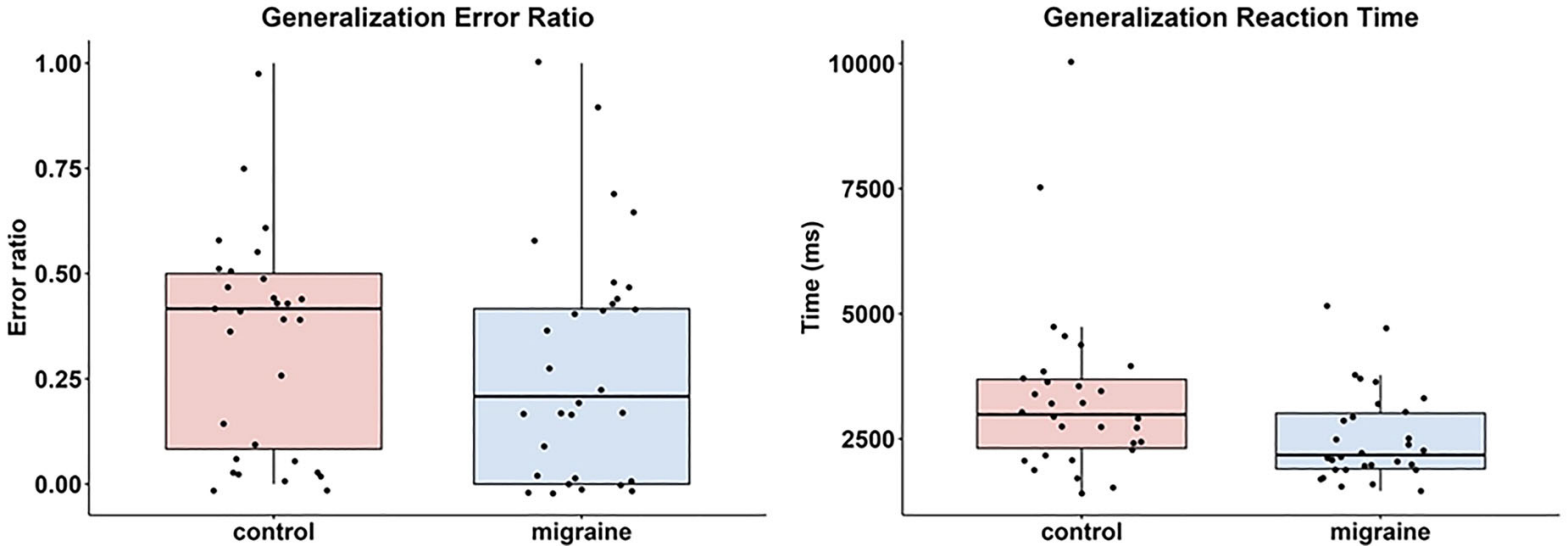
Boxplot comparisons of generalization performance in the test phase of the SoundPolygon test in the healthy control group (*n* = 30) and the migraine group (*n* = 30). No significant differences were found between the two groups in generalization error ratios (right; Mann–Whitney *U* test: *p* = 0.464; *r* = 0.094). Significantly shorter generalization reaction times were found in the migraine patient group (left; Mann–Whitney *U* test: *p* = 0.024; *r* = 0.291). Plotting conventions are the same as described in Figure 5.

There was no significant difference in the generalization error ratios (GER) between the two groups (*U* = 400.00, *p* = 0.464, *r* = 0.094). The median GER was 0.208 in the patient group (Q1: 0.000–Q3: 0.417) and 0.417 in the control group (Q1: 0.083–Q3: 0.500). Generalization reaction times (GRT) were significantly shorter in the patient group compared to the control group (*U* = 297.00, *p* = 0.024, *r* = 0.291). The median GRT in the patient group was 2178 ms (Q1: 1883 ms–Q3: 3039 ms), and in the control group, it was 2989 ms (Q1: 2281 ms–Q3: 3710 ms) (Figure 14).

## 4 Discussion

To our knowledge, this is the first study to investigate the influence of semantic content of consequent visual stimuli on audiovisual associative learning performance in patients with migraine. We employed two different audiovisual tests: one that used visual stimuli with higher semantic content (SoundFace test) (Eördegh et al. 2019), and another that used visual stimuli with lower semantic content (SoundPolygon test) (Tót et al. 2022). In both tests, the same four distinct auditory stimuli were used as antecedents. Our results demonstrated that performance strongly depends on the semantic content of the visual stimuli; those with higher semantic content elicited better performance in audiovisual associative equivalence learning in the studied adult migraine population. On the other hand, the cognitive performance of the migraine patients was not different from that of the matched healthy control group in audiovisual learning using consequent visual stimuli with lower semantic content.

A more detailed examination of the results revealed significant differences in the acquisition phase: participants performed significantly better in the SoundFace test than in the SoundPolygon test in terms of the number of acquisition trials (NAT), acquisition error ratios (AER), and acquisition reaction times (ART). This finding aligns with our previous results comparing the same two audiovisual learning tests in healthy controls (Tót et al. 2022), who also performed significantly better in the SoundFace test than in the SoundPolygon test. We conclude that the same trend persists: participants perform significantly worse when the consequent visual stimuli contain less semantic content.

Similar to the acquisition phase, significant differences were found in test‐phase performance between the two audiovisual tests. Both the error ratios and reaction times for retrieval (RER, RRT) and generalization (GER, GRT) were significantly lower and shorter in the SoundFace test than in the SoundPolygon test. This finding is consistent with the trend previously observed in a healthy population (Tót et al. 2022).

The superior performance observed in the SoundFace task is likely not attributable to a single property of the stimuli but rather to several complementary factors. One of these factors may be the higher semantic content of the facial stimuli. Previous findings applying the same visual stimuli demonstrated that visual semantic content substantially contributes to associative equivalence learning, suggesting that richer semantic representations facilitate the formation and retrieval of associative relationships (Tót, Harcsa‐Pintér, et al. 2025). In addition, faces constitute a biologically and socially highly relevant stimulus category (Adolphs 2009; Tsao and Livingstone 2008) that is processed by specialized neural systems, including the occipital face area, fusiform face area, and superior temporal sulcus (Duchaine and Yovel 2015; Haxby et al. 2000; Kanwisher et al. 1997; Pitcher et al. 2007), which may facilitate associative encoding. Faces are also processed predominantly configurally, allowing more efficient discrimination than abstract geometric figures (Maurer et al. 2002; Piepers and Robbins 2012). Finally, the greater perceptual and categorical distinctiveness of individual faces, compared with the relatively similar polygon stimuli, may further facilitate encoding and retrieval during associative learning (Burton et al. 2005; Valentine 1991). Although the present study cannot determine the relative contribution of these mechanisms, they may collectively contribute to the superior performance observed with facial stimuli.

The present study also includes the comparison of performance between migraineurs and matched controls in the audiovisual associative equivalence test with visual stimuli that contain less semantic content (SoundPolygon test). Our results revealed that migraine patients do not show impaired performance compared to healthy controls in this specific learning task.

Significant differences were not found between the two groups in the acquisition phase in terms of the number of trials (NAT), error ratios (AER), and reaction times (ART). Test‐phase performance revealed significantly shorter reaction times in generalization (GRT) in the patient group. No significant differences were observed in retrieval error ratios and reaction times (RER, RRT), and generalization error ratios (GER). This preserved audiovisual learning with consequent visual stimuli with lower semantic content is similar to recent findings from audiovisual learning with consequent visual stimuli with higher semantic content (SoundFace test), where acquisition performance was even better in the migraine patient population (Tót et al. 2024).

In a recent study investigating purely visual associative learning in patients with migraine (Tót, Eördegh, et al. 2025), we found that participants performed better—though not to a statistically significant extent—in the test which used visual stimuli with higher semantic content (RAET or Face‐Fish test) (Myers et al. 2003), compared to the corresponding test with lower semantic content visual stimuli (Polygon test) (Eördegh et al. 2022). Only reaction times were significantly longer in the Polygon test, suggesting a performance‐impairing effect of visual stimuli with lower semantic content in equivalence learning. This subtle impairing effect appears to be more pronounced in audiovisual learning, as evidenced by the current findings. All metrics for patients with migraine—number of acquisition trials, performance, and reaction times across all phases—were significantly worse in the SoundPolygon test compared to the SoundFace test.

Previous studies in adults with migraine showed that performance in the acquisition and generalization phases of the RAET was poorer compared to that of an age‐, gender‐, and education‐level‐matched control population (Giricz et al. 2021; Öze et al. 2017). In contrast, a recent study found that patients with migraine performed significantly better than matched controls in the acquisition phase of audiovisual associative learning when visual consequent stimuli with higher semantic content were used (SoundFace test), although no significant difference was found in the test phase. These findings suggested the presence of possible compensatory mechanisms in audiovisual learning among patients with migraine compared to visually guided associative learning (Tót et al. 2024; Tót, Eördegh, et al. 2025). This interpretation is also consistent with neuroimaging results suggesting that preserved behavioral performance in migraine may be accompanied by altered recruitment of neural networks during visuospatial processing (Messina et al. 2021). Previous population‐based results have also reported preserved or even better cognitive performance among individuals with migraine in certain cognitive domains (Wen et al. 2016), suggesting that migraine is not uniformly associated with cognitive impairment.

One possible explanation for the results of the present study is the engagement of compensatory cognitive processes during audiovisual associative learning (Tót et al. 2024; Tót, Eördegh, et al. 2025), as the same trend was observed in both the migraine group and the healthy control group (Tót et al. 2022); however, the present behavioral data alone do not permit conclusions regarding the underlying neural mechanisms. In both groups, participants performed better in the audiovisual associative learning task that used consequent visual stimuli with higher semantic content. The two sets of visual consequent stimuli were intentionally designed to differ markedly in order to highlight the facilitating effect of stimuli with higher semantic content—specifically, faces in the SoundFace test—compared to low‐semantic‐content stimuli, such as polygons in the SoundPolygon test.

Another possible explanation for why patients with migraine performed better than matched controls in the SoundFace test is sensory hypersensitivity (Tót et al. 2024). Hypersensitivity in migraine can manifest across multiple sensory modalities and may lead to cross‐sensitization (Schwedt 2013). The same mechanisms might also contribute to preserved performance among patients with migraine in the SoundPolygon test compared to healthy controls.

Additionally, preserved performance in migraine can be explained by cognitive processing strategies differing from those of healthy individuals. Recent neuroimaging results suggest enhanced implicit statistical learning in migraine accompanied by altered BOLD signal variability, indicating that preserved or even enhanced learning performance may arise from differences in neural processing rather than from impairment alone (Kocsis et al. 2026). However, alternative explanations, including differences in attentional strategies, motivational factors, or the greater salience of stimuli with higher semantic content, cannot be excluded on the basis of the present behavioral data.

One limitation of this study is the relatively small sample size. Although an a priori sample size estimation was performed, the between‐group analyses did not reach the planned sample size. Therefore, smaller between‐group effects may have remained undetected, and these findings should be interpreted with appropriate caution. However, it should not be forgotten that this was a well‐defined migraine population without any neurological or psychiatric comorbidities, and the patients were free from interval migraine therapy. Nevertheless, it would be beneficial to repeat the investigation with a larger, independent sample of patients and matched controls. Another limitation of the study concerns the sex distribution, which is skewed toward females due to the nature of migraine (Vetvik and MacGregor 2017). Migraine most commonly affects adult women; however, this likely did not bias our results, as each participant's performance was compared across the two tests individually. In this and our previous studies (Eördegh et al. 2019; Tót et al. 2022), we aimed to establish two clear endpoints representing low and high semantic content in the consequent visual stimuli. The SoundFace test used visual stimuli with high semantic content, while the SoundPolygon test used clearly simpler stimuli with minimal semantic content. Future research may benefit from refining the range of visual consequent stimuli within this spectrum. As noted in the Methods section, all data were collected at least three days after participants’ last reported migraine attack. However, we have no information regarding the timing of the next attack following testing. Therefore, we cannot rule out the possibility that some participants were in a preictal rather than interictal phase during the performance of the learning tests (Giffin et al. 2016; Giffin et al. 2003; Omland 2024; Peng and May 2020; Schulte and May 2016). A further limitation of the present study is that only behavioral performance measures were obtained. Consequently, although compensatory mechanisms represent one possible interpretation of the findings, the present data do not provide direct evidence for altered neural processing. Future studies combining associative learning tests with electrophysiological or neuroimaging techniques will be necessary to determine whether compensatory neural mechanisms underlie the preserved behavioral performance observed in migraine.

## 5 Conclusion

In summary, this study investigated how the semantic content of visual stimuli influences audiovisual associative learning in patients with migraine. Two tests were used, employing visual consequents with either higher semantic content (SoundFace) or lower semantic content (SoundPolygon), and performance was compared between patients and healthy controls in the SoundPolygon test. A similar pattern of performance across the two tests was observed in migraine patients and healthy individuals. The simpler visual consequents elicited reduced audiovisual learning performance in migraine patients as well. Additionally, no impaired learning performance was found between patients and matched controls in the SoundPolygon test, where visual consequents with lower semantic content were applied. Interestingly, in our recent study, migraine patients outperformed healthy adults in the SoundFace test, which involved visual consequents with higher semantic content in audiovisual learning. Taken together, these findings indicate preserved audiovisual associative learning in patients with migraine despite the use of visual stimuli with lower semantic content. One possible explanation is the engagement of compensatory cognitive processes; however, alternative explanations, including differences in attentional strategies, sensory hypersensitivity, motivational factors, or stimulus salience, cannot be excluded. Future electrophysiological and neuroimaging studies are needed to clarify the mechanisms underlying these behavioral findings.

## Author Contributions

**Noémi Harcsa‐pintér**: Writing – original draft, Writing – review and editing, visualization, investigation, formal analysis. **Gábor Braunitzer**: conceptualization, writing – original draft, writing – review and editing, methodology. **Kálmán Tót**: writing – review and editing, visualization, formal analysis, investigation, data curation. **Anett Csáti**: investigation, writing – review and editing. **János Tajti**: investigation, writing – review and editing. **Adél Papp**: investigation, formal analysis, writing – review and editing. **Balázs Bodosi**: conceptualization, software, writing – review and editing, methodology. **Attila Nagy**: conceptualization, writing – original draft, writing – review and editing, funding acquisition, methodology, project administration, supervision, resources. **Gabriella Eördegh**: conceptualization, investigation, writing – review and editing, methodology.

## Funding

Open access funding is provided by the University of Szeged. This work was supported by the SZTE SZAOK‐KKA‐SZGYA Grant No. 2023/5S479.

## Ethics Statement

This study was approved by the Regional Research Ethics Committee for Medical Research at the University of Szeged, Hungary (27/2020‐SZTE, NNGYK/31295‐6/2024). All participants provided written informed consent.

## Patient Consent Statement

All participants provided written informed consent.

## Conflicts of Interest

The authors declare no potential conflicts of interest with respect to the research, authorship, and/or publication of this article.

## Data Availability

The data that support the findings of this study are available from the corresponding author upon reasonable request.
